# The Influence of Virtual Forest Walk on Physiological and Psychological Responses

**DOI:** 10.3390/ijerph182111420

**Published:** 2021-10-29

**Authors:** Emad Alyan, Theo Combe, Dayang Rohaya Awang Rambli, Suziah Sulaiman, Frederic Merienne, Nadia Diyana Mohd Muhaiyuddin

**Affiliations:** 1 Department of Computer and Information Sciences, University Teknologi PETRONAS, Seri Iskandar 32610, Perak, Malaysia; combetheo@gmail.com (T.C.); suziah@utp.edu.my (S.S.); 2 Arts et Metiers Institute of Technology, LISPEN, HESAM Université, F-71100 Chalon-sur-Saône, France; frederic.merienne@ensam.eu; 3 School of Multimedia Technology and Communication, University Utara Malaysia, Sintok 06010, Kedah, Malaysia; nadia.diyana@uum.edu.my

**Keywords:** virtual reality (VR), stress, forest therapy, psychophysiological response

## Abstract

The authors of this paper sought to investigate the impact of virtual forest therapy based on realistic versus dreamlike environments on reducing stress levels. Today, people are facing an increase in stress levels in everyday life, which may be due to personal life, work environment, or urban area expansion. Previous studies have reported that urban environments demand more attention and mental workload than natural environments. However, evidence for the effects of natural environments as virtual forest therapy on stress levels has not yet been fully explored. In this study, a total of 20 healthy participants completed a letter-detection test to increase their stress level and were then randomly assigned to two different virtual environments representing realistic and dreamlike graphics. The participants’ stress levels were assessed using two physiological methods that measured heart rate and skin conductance levels and one psychological method through the Profile of Mood States (POMS) questionnaire. These indicators were analyzed using a sample *t*-test and a one-way analysis of variance. The results showed that virtual forest environments could have positive stress-relieving effects. However, realistic graphics were more efficient in reducing stress. These findings contribute to growing forest therapy concepts and provide new directions for future forest therapy research.

## 1. Introduction

Stress is a situation that develops when an individual perceives a mismatch between the demands of a circumstance and his/her biological, psychological, or social resources [1]. According to the literature, prolonged stress can negatively and severely influence health [2]. Stress is linked to a variety of mental illnesses and cardiovascular problems [3,4]. The environment plays an essential role in our capacity to deal with stress factors. The environment can improve or decrease people’s coping strategies in the face of stressful situations [5,6,7]. For instance, the urban environment is visually complex and requires more attention than natural environments [8]. Therefore, spending time in a non-urban area, where attention is in its natural environment, can help to alleviate urban stress. From a psychological perspective, Ulrich, et al. [9] presented the stress relief theory, which claims that exposure to nature can relieve stress and directly impact cognitive restoration.

The association between interaction with forests and public health has long been argued and extensively demonstrated. The literature suggests that forests may improve health status more than urban environments [10,11]. Individuals exposed to stimuli derived from a forest can experience preventive medical effects, acquire physiological relaxation, and boost immune system weakness [12,13]. The term “forest therapy” refers to a form of alternative therapy that helps to strengthen the immune system and enhance mental health by attaining a state of physiological relaxation [10,14].

There have been many studies on the impacts of forest exposure, mainly focusing on the psychological and physiological implications of forest therapy. It has been claimed that visiting forests can reduce one’s cortisol levels, blood pressure, respiratory rate, and heart rate [12,15,16], which can contribute to alleviating mental symptoms (such as anxiety, nervousness, stress, and fatigue) and improve mood and cognitive states [17,18,19]. Another nature-based well-being practice called forest bathing or “Shinrin-yoku” has been proven to reduce undue stress and potential burnout [20]. According to several studies, visiting a forest can prevent disease and strengthen immunity [21].

Despite significant evidence demonstrating the benefits of forest visits, it is difficult for people in urban cities to regularly visit forests and experience their positive effects. However, instead of stepping outside, an emerging technology based on virtual reality (VR) has been developed to provide an immersive environment to simulate interactions with real nature. For example, a head-mounted display (HMD) can be used to give a feeling of deep immersion that is very close to the real situation [22]. Recently, VR systems have rapidly grown and confirmed their effectiveness in mimicking natural forests for applications of healthcare and psychology disorders [23] such as stress [24], anxiety [25], and social phobia [26]. Different psychiatric patients have reported that VR environments can alter their anxiety, cognition, depression, and social functions [27]. For example, Ahmadpour, Keep, Janssen, Rouf and Marthick [25] suggested that VR could be a practical solution for pain and anxiety management in various medical procedures. Furthermore, other studies have demonstrated the ability of VR to reduce posttraumatic stress disorder (PTSD) symptoms [23] and suggested its use in treating PTSD. Although few studies have examined the impact of the forest environment on human physiological and mental health [24,28], the research in this field is still in its infancy.

Consequently, the authors of the present study sought to contribute new knowledge to forest therapy and to examine the physiological stress level resulting from the effects of exposure by measuring skin conductance and heart rate. Previous studies have linked declines in skin conductance [29,30,31] and heart rate [24,32] to decreased stress levels. Our aim was to quantify participants’ moods after viewing different forest environments used to reduce psychological stress through a well-standardized scale—the Profile of Mood States (POMS) questionnaire, which is extensively used in many situations, including past studies that assessed mood in people exposed to various virtual forest environments [24,33].

## 2. Materials and Methods

### 2.1. Demographics

The sociodemographic information of the participants in this research is reported in Table 1. The authors of this study enrolled 20 healthy university students comprised of 10 males and 10 females who had normal vision or corrected normal vision and no history of cardiovascular disease, cognitive impairment, or psychiatric disorders. The average age of all participants was 21.8 ± 2.2 years old. The participants were instructed to obtain plenty of sleep (>8 h) and to avoid coffee, smoking, drugs, alcohol, and strenuous physical activity on the experiment day.

### 2.2. Study Site and Stimuli

#### 2.2.1. Stress Induction Method

**Figure 1:**
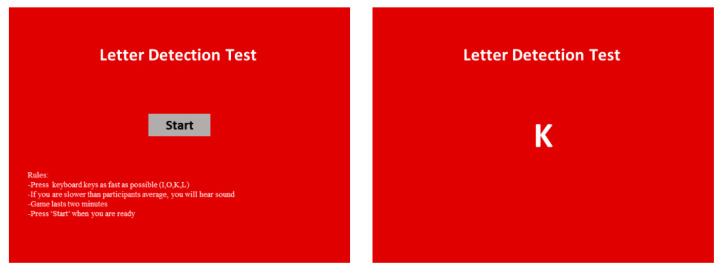
Letter-detection test.

We developed a computerized task based on the letter-detection test (LDT) to provoke stress in the lab environment [34]. The task involved a sequence of letters displayed on a computer screen, as shown in Figure 1. Participants had to press the correct keyboard key as fast as possible. This was a challenging task because the letters were moving so quickly. Additionally, participants received negative auditory feedback when they pressed the wrong key. The duration between the two actions shortened every 30 s (from 1 s between two letters to 0.25 s), and a stressful noise occurred when the remaining time was less than a minute.

#### 2.2.2. Design and Equipment

**Figure 2:**
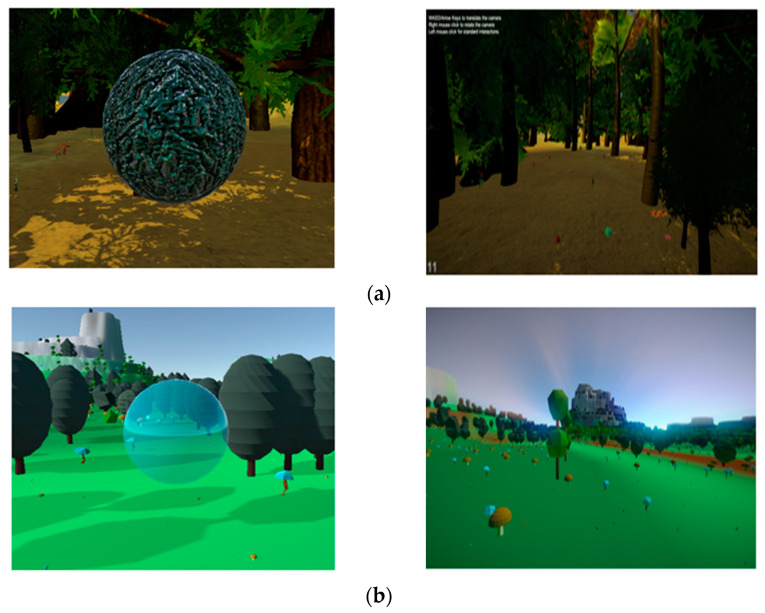
Representation of designed virtual environments: (**a**) realistic and (**b**) dreamlike.

To examine exposure to the forest environment, we created two virtual environments, one realistic and the other dreamlike. The dreamlike environment (DE) can be described as an illusory atmosphere that lacked reality compared to the realistic environment (RE) (refer to Figure 2). The virtual environments were specifically developed to help reduce the physiological and psychological stress via improved engagement provided through virtual immersion in a forest environment with bubbles that disappeared when touched. These bubbles were incorporated into the environment to capture participants’ attention and motivate them to complete the task. During the experiment, the participants had to explore the environments by touching bubbles using a touchpad, looking around, and hearing various sounds of nature such as birds and waterfalls. Both environments, realistic and dreamlike, had the same terrain, interactions, and sounds.

The assets for the virtual environments were designed in 3DS Max and compiled in Unity using Visual Studio. VR equipment included the HTC Vive with a 1440 × 1600 pixel-per-eye display at 110 degrees tethered viewing angle.

### 2.3. Measures

#### 2.3.1. Physiological Index

The heart rate (HR) and skin conductance level (SCL) have been utilized as biomarkers to indicate short-term stress response by non-invasively monitoring individuals’ autonomic nervous system reactions, particularly when comparing pre and post-experiment data. Here, the SCL was sampled at 32 Hz using sensors (Model SA9309M, Thought Technology) attached to the index and ring fingers to measure skin conductivity in MicroMho (0–30 MΩ) because MicroMho rises in response to an increase in anger-related arousal [35]. Additionally, the HR was recorded at a sampling rate of 32 Hz through a blood volume pulse (BVP) sensor (Thought Technologies, model SA9308M) placed on the index finger to assess heart rate in beats per minute (bpm). That is, an infrared sensor was used to detect the participant’s blood cell reflection. The signal reflected by each blood cell was processed and used to compute the participants’ cardiovascular change based on their pulse and blood circulation speed. According to previous research, variations in the BVP amplitude and SCL value imply sympathetic arousal that increases under stress and decreases in a pleasant environment [36,37].

#### 2.3.2. Psychological Index

The POMS questionnaire was utilized to monitor mood changes, particularly participants’ psychological stress [38]. Many studies have demonstrated that POMS is a validated self-report survey that possesses adequate psychometric properties in clinical psychology, psychotherapy, and medicine to assess psychological distress and distinct subjective mood states. It is sensitive to mood-related emotional changes. For instance, Zhang, et al. [39] used POMS to examine people’s psychological response to an enclosed wooden area. Yu, Lee, and Luo [11] also used this scale to investigate the psychological effects of a VR forest and an urban area.

This scale consists of 40 options that define subjective feelings in six different mood states (*T*: tension; *D*: depression; *A*: anger; *F*: fatigue; *C*: confusion; and *V*: vigor). A 5-point Likert scale, ranging from 0 (Not at all) to 4 (Extremely), was used for each item. The higher the score, the more pronounced the participant’s emotion was. After the experiment, participants were asked to fill out the scale based on their emotional states. The total mood disturbance (*TMD*) was computed by summing the negative mood state scales and subtracting *V* (a positive mood state scale):

(1) TMD=(T+D+A+F+C)−(V)

In which an increase in the mean value of *TMD* could imply a deterioration in participants’ mood.

### 2.4. Experimental Procedure

Participants were first briefly introduced to the experiment to allow them to become accustomed to the environment. This included the purpose and stages of the experiment, the hardware to be used, and the questionnaires to be filled. Before the experiment, participants were required to fill out a consent form for participation. During the experiment, participants were initially measured as baselines for HR and SCL to ensure they were in normal states and to allow them to become practically accustomed to the device.

**Figure 3:**
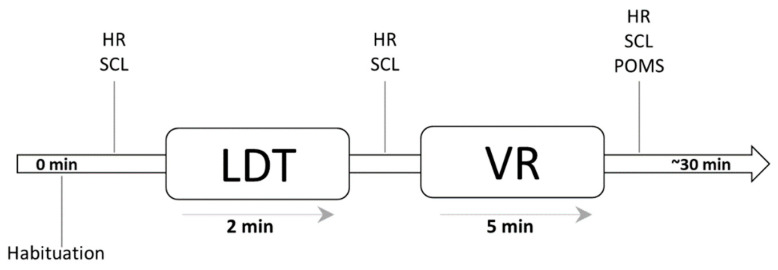
Timeline of the experimental process (LDT = letter-detection test; VR = virtual reality; HR = heartrate; SCL = skin conductance level; POMS = Profile of Mood States).

The LDT was then conducted, and the participants were instructed to perform a 2 min letter detection task. The LDT was designed to induce stress levels in every participant. After the LDT, the pre-testing of HR and SCL was conducted, reflecting the participants’ stress levels prior to the VR experiment. For 5 min, participants were then randomly assigned to either a realistic or a dreamlike virtual environment (RE or DE, respectively), assuming that this could reduce stress to some extent. This was followed by the post-tests, including the POMS and previous tests (of HR and SCL), demonstrating stress levels after experiencing the virtual forest environments. The timeline of the process is illustrated in Figure 3.

### 2.5. Statistical Analysis

A statistical analysis of the experimental data was carried out using MATLAB and SPSS version 24.0. (SPSS Inc., Chicago, IL, USA). Two analytical methods were used: (1) for the HR and SCL data before and after the LDT, related paired *t*-tests were carried out to verify whether the LDT played a role in increasing stress levels, and (2) for the same indicators (HR and SCL), the differences between the pre- and post-test for RE and DE were used to determine whether these environments played a role in relieving stress. Additionally, a one-way analysis of variance (ANOVA) with Bonferroni correction was used to evaluate the effects of the two tested environments (RE and DE) on relieving psychological stress levels. The effect size was calculated using Cohen’s d and partial eta squared (η_p_^2^). The significance levels were set at * *p* < 0.05, ** *p* < 0.01, and *** *p* < 0.001. The data are presented as means and standard deviations (SDs).

## 3. Results

### 3.1. Physiological Response to Virtual Environments

**Figure 4:**
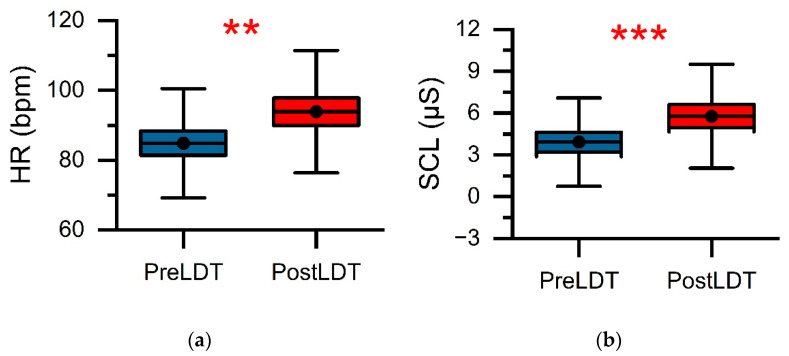
Mean values of (**a**) HR and (**b**) SCL for pre- and post-LDT. The error bars indicate the SD, and the red stars indicate significant differences (** *p* < 0.01 and *** *p* < 0.001).

Figure 4 depicts the mean levels of HR and SCL for the twenty participants, along with the measurements taken before and instantly after the LDT. Paired *t*-tests were conducted to examine the change in HR and SCL levels associated with the LDT (refer to Table 2). The results showed that for participants who completed the LDT, their HR (mean = 93.9, SD = 17.5) and SCL (mean = 5.8, SD = 3.7) levels were higher than they were at baseline (HR: mean = 84.9, SD = 15.6; SCL: mean = 3.9, SD = 3.2). Both physiological indexes (HR: *t*(1,19) = 3.5, *p* < 0.01, *d* = 0.54; SCL: *t*(1,19) = 7.3, *p* < 0.001, *d* = 0.55) showed statistically significant differences between pre- and post-LDT.

**Figure 5:**
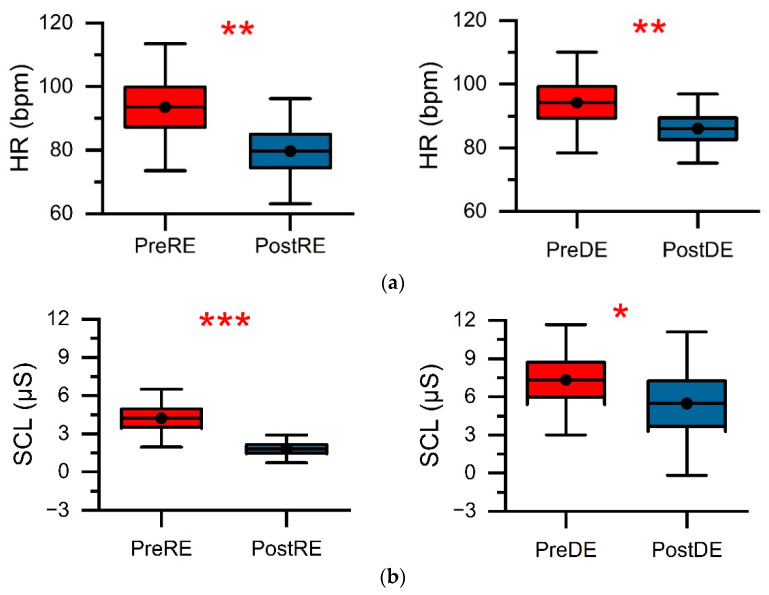
Average values of (**a**) HR and (**b**) for pre- and post-test of (**left**) RE and (**right**) DE. The error bars indicate the SD, and the red stars indicate significant differences (* *p* < 0.05, ** *p* < 0.01, and *** *p* < 0.001).

Furthermore, the HR and SCL data of the pre- and post-test of the real and dreamlike environment groups were assessed using paired *t*-tests. The pre- and post-test data for both types of virtual environments showed statistically significant differences. Figure 5a,b—for the HR and SCL data, respectively—indicate that the RE and DE were able to help reduce the stress levels of the participants. However, the RE (HR: *t*(1,9) = 3.3, *p* < 0.01, *d* = 0.75; SCL: *t*(1,9) = 5.3, *p* < 0.001, *d* = 1.20) was highly significant in reducing stress levels compared to the DE (HR: *t*(1,9) = 3.1, *p* < 0.01, *d* = 0.59; SCL: *t*(1,9) = 2.5, *p* < 0.05, *d* = 0.33).

### 3.2. Psychological Response to Virtual Environments

**Figure 6:**
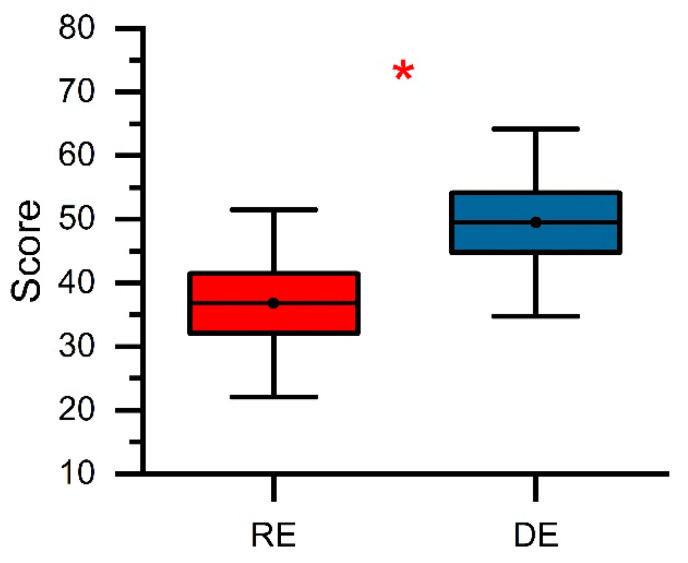
The total mood disturbance (*TMD*) for the RE and DE. Interpretation: red (*) indicates *p* < 0.05.

The mean *TMD* scores of both virtual environments, RE and DE, are illustrated in Figure 6, while Table 3 summarizes the statistical results for both environments. The results show that the RE (mean = 36.8, SD = 14.8) was significantly different from the DE (mean = 49.5, SD = 14.8). That is, the *TMD* was significantly decreased (*t*(1,19) = 1.9, *p* < 0.05, *d* = 0.86) in the RE compared to the DE, where a high *TMD* score indicates a good emotional condition.

**Figure 7:**
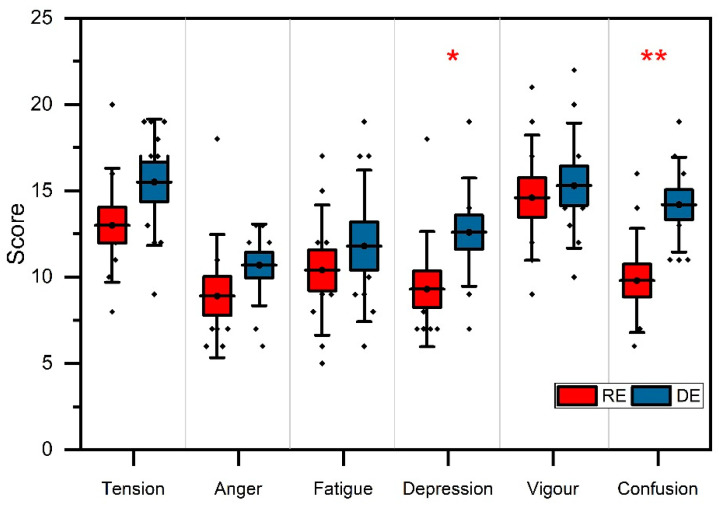
Comparison of POMS subscales for the RE and DE. Interpretation: red (*) and (**) indicate *p* < 0.05 and *p* < 0.01, respectively. The error bars represent the SD.

In addition, a plot was created for the RE and DE of POMS, as shown in Figure 7. The one-way ANOVA test demonstrated significant differences in the total POMS values and its subscale (refer to Table 4). The POMS scores of all subscales were more decreased in the RE compared to the DE. For example, the depression and confusion decreased by 3.3 and 4.4, respectively, and showed significant differences (*D*, *F*(1,18) = 5.2, *p* < 0.05, η_p_^2^ = 0.22; *C*, *F*(1,18) = 11.7, *p* < 0.01, η_p_^2^ = 0.39). In contrast, tension, anger, fatigue, and vigor decreased by 2.5, 1.8, 1.4, and 0.7 points, respectively, showing no significant differences.

## 4. Discussion

The authors of this study evaluated the restorative states of participants’ psychological and physiological stress before and after viewing realistic and dreamlike environments with VR devices. The results indicated that the use of VR-based forest environments could be useful in aiding mental well-being. Importantly, we found that the use of VR led to significant decreases in participants’ psychological and physiological stress levels.

### 4.1. The Impact of Virtual Forest Walk on Physiological Responses

One of the most remarkable outcomes in this study was the significant association between virtual forest environments and positive psychological responses. The physiological measurements (HR and SCL) demonstrated that participants in the virtual forest environments reported more significant positive psychological impacts. For example, upon exposure to realistic and dreamlike virtual environments, the mean values of HR decreased by approximately 14.76% and 8.70%, respectively, and the SCL decreased by 57.14% and 24.66%, respectively. The participants in the virtual forest environments with dreamlike scenes showed significant positive effects to a certain extent (HR: *p* < 0.01; SCL: *p* < 0.05), though not as significant as with realistic scenes (HR: *p* < 0.01; SCL: *p* < 0.001).

The results showed that a realistic environment could substantially positively impact sympathetic activity, resulting in a quicker recovery from stressful situations. These findings were generally consistent with past research that discussed physiological response when exposed to forest environments [24] and the positive effect of rural landscapes on stress [40]. However, the reported high significance in the realistic environment might have been more related to the greenery of scenes compared to the dreamlike environment, which was consistent with previous research findings on the use of green environments to mitigate stress [40,41]. This potential of VR has been validated by other studies that have reported its capability to reduce pain, fear, and anxiety associated with needle-related procedures [42] and others that have revealed its positive impact on psychiatric disorders [23].

### 4.2. The Impact of Virtual Forest Walk on Psychological Responses

From the standpoint of psychological response, this study also showed the impact of realistic and dreamlike forest environments on stress recovery. The measured *TMD* data were analyzed with the appropriate two-sample *t*-test, which proved that the realistic and dreamlike environments had statistically significant different effects on psychological stress levels. The *TMD* values were significantly decreased (*p* < 0.05) when participants were exposed to the realistic environment compared to the dreamlike environment. This suggested that the realistic environment could relieve stress levels to a better extent than the dreamlike environment, supporting the results of the physiological measures.

Further analysis based on one-way ANOVA showed the differences between the realistic and dreamlike environments were significant in two subscales (depression: *p* < 0.05; confusion: *p* < 0.01) of the POMS. This variation could have been due to a variety of natural factors in the VR of the two environments. This result is compatible with our physiological results and those of several studies related to the effects of forest environments on, e.g., stress relief, emotional situation adaptation, the positive association between health parameters and natural environments, and the positive impact of the forest on the activity of the nervous system (such as autonomic activity) [33,40,41,43,44]. Generally, walking is connected with therapeutic effects, and most people consider it a beneficial activity [45]. Past research studies have specifically looked at the impact of VR walking on well-being outcomes, and evidence suggests that even small amounts of outdoor exercise could improve self-esteem and mood [46]. The results of our study imply that VR nature experiences may be an adequate substitute for individuals who cannot visit natural areas for any reason.

### 4.3. Limitations and Directions for Future Research

This study had some limitations that could be addressed in future research. First, the authors of this study only investigated the stress-relieving impact of two forest environments. A greater variety of real forest environments could be considered in future research. Researchers should explore the interaction of environmental characteristics such as climate and lighting with psychophysiological effects. Second, to achieve consistency in the data, future research should consider the participants’ prior experience with VR. Third, the authors of this study observed that different forest environments had various stress recovery effects. However, the underlying causes of the variations, potentially including beauty, naturalness, and peace of mind, were not discovered. That is, the internal mechanism responsible for the various stress recovery effects was not identified. Fourth, in addition to the relatively small sample size used in the study, the target population was undergraduate students, so our results cannot be generalized to the general public who may participate in forest therapy. Fifth, more physiological measures such as salivary alpha-amylase and cortisol levels could be considered in future studies to validate the HR and SCL measures. In the future, heart rate variability (HRV) and neuroimaging techniques such as EEG, fNIRS, and fMRI should be added to improve the objectivity of research results.

## 5. Conclusions

The authors of this study evaluated the impacts of two virtual forest environments on participants’ moods and psychophysiological responses by providing objective evidence through HR, SCL, and POMS measures. The findings demonstrated that the forest environment is one of the contributing factors to the psychophysiological impacts in forest therapy. However, diverse types of forest environments may have varying impacts on stress reduction. The forest environments used in this study had the effect of relieving stress, especially the realistic forest environment, which played a more significant role in relieving physiological stress (HR: *p* < 0.01; SCL: *p* < 0.001) than the dreamlike environment (HR: *p* < 0.01; SCL: *p* < 0.05). In this context, the realistic forest environment was also more effective at rapidly alleviating psychological stress (POMS: *p* < 0.05) than the dreamlike environment. These findings may pave the way for future research into forest therapy. Modern life is becoming increasingly stressful, so understanding how to maximize the benefits of virtual forest environments is essential.

## Figures and Tables

**Table 1:** List of the sociodemographic characteristics of the study participants.

	Total Sample	
	RE	DE
N	10	10
Gender		
Male	5	5
Female	5	5
Age Group		
18–25	23 (1.1)	20.6 (2.4)
Education		
University Degree	10	10

**Table 2:** The statistical results of physiological indexes (HR and SCL) for pre- and post-test of the LDT, RE, and DE.

Index	Pre		Post		Paired *t*-Test		
	Mean	SD	Mean	SD	*t*-Value	*p*-Value	Cohen’s *D*
**HR**							
LDT	84.9	15.6	93.9	17.5	3.5	0.001 **	0.54
RE	93.5	20.0	79.7	16.5	3.3	0.005 **	0.75
DE	94.2	15.8	86.0	10.8	3.1	0.006 **	0.59
**SCL**							
LDT	3.9	3.2	5.8	3.7	7.3	0.000 ***	0.55
RE	4.2	2.3	1.8	1.1	5.3	0.000 ***	1.20
DE	7.3	4.3	5.5	5.6	2.5	0.016 *	0.33

SD: standard deviation, HR: heart rate, SCL: skin conductance level, *: *p* < 0.05, **: *p* < 0.01, ***: *p* < 0.001.

**Table 3:** The statistical results of *TMD* score for the RE and DE.

VR	Mean	SD	Two-Sample *t*-Test		
			*t*-Value	*p*-Value	Cohen’s *D*
RE	36.8	14.8	1.9	0.035 ^*^	0.86
DE	49.5	14.8			

*: *p* < 0.05.

**Table 4:** Statistics for the POMS subscale comparison between the RE and DE.

POMS Subscale	RE		DE		One-Way ANOVA		
	Mean	SD	Mean	SD	F-Value	*p*-Value	η_p_^2^
Tension	13.0	3.3	15.5	3.7	2.6	0.126	0.13
Anger	8.9	3.6	10.7	2.4	1.8	0.200	0.09
Fatigue	10.4	3.8	11.8	4.41	0.6	0.455	0.03
Depression	9.3	3.3	12.6	3.1	5.2	0.035 *	0.22
Vigor	14.6	3.6	15.3	3.6	0.2	0.671	0.01
Confusion	9.8	3.0	14.2	2.7	11.7	0.003 **	0.39

RE: realistic environment, DE: dreamlike environment, SD: standard deviation, *: *p* < 0.05, **: *p* < 0.01.

## Data Availability

The data presented in this study are available on request from the corresponding author.
